# Always Give Credit Where Due

**Published:** 1897-11

**Authors:** 


### ALWAYS GIVE CREDIT WHERE DUE.

THE *Catechism on Tuberculosis* issued by the Secretary of the New York State Board of Health, and reproduced in full in the *American Cultivator and Country Gentleman*, appears to be a complete reproduction of Circular No. 4 of the State Live-stock Sanitary Board of Pennsylvania, without any credit being given the latter board for the same. We have nothing but praise for its contents and advice, and would wish for it the utmost publicity all over the land; it is educational, instructive, impartial, and fair in all its replies and deductions; but when put out by boards of sister States, it would seem only the most meagre justice to give credit to its original production and source from which it emanated, and not impose it upon periodicals of the Empire State as an original production of the Empire State Board of Health.

